# Comparative genomics of the T cell receptor μ locus in marsupials and monotremes

**DOI:** 10.1007/s00251-023-01320-w

**Published:** 2023-09-25

**Authors:** K. A. Morrissey, M. R Stammnitz, E. Murchison, R. D. Miller

**Affiliations:** 1 Center for Evolutionary and Theoretical Immunology, Department of Biology, University of New Mexico Albuquerque, NM, USA; 2 Centre for Genomic Regulation (CRG), The Barcelona Institute of Science and Technology, Barcelona, Spain; 3 Transmissible Cancer Group, Department of Veterinary Medicine, University of Cambridge, Cambridge, UK

**Keywords:** T cell receptor genes, marsupials, monotremes

## Abstract

T cells are a primary component of the vertebrate adaptive immune system. There are three mammalian T cell lineages based on their T cell receptors (TCR). The αβ T cells and γδ T cells are ancient and found broadly in vertebrates. The more recently discovered γμ T cells are uniquely mammalian and only found in marsupials and monotremes. In this study, we compare the TCRμ locus (*TRM*) across the genomes of two marsupials, the grey short-tailed opossum and Tasmanian devil, and one monotreme, the platypus. These analyses revealed lineage specific duplications, common to all non-eutherian mammals described. There is conserved synteny in the *TRM* loci of both marsupials but not in the monotreme. Our results are consistent with an ancestral cluster organization which was present in the last common mammalian ancestor which underwent lineage specific duplications and divergence among the non-eutherian mammals.

## Introduction

Critical to the vertebrate adaptive immune response are the T and B lymphocytes which use antigen specific receptors, the T cell receptors (TCR) and immunoglobulins (Ig), respectively, to recognize everything from pathogens to cancer cells. There are three lineages of T cells in mammals, as defined by the composition of their TCR heterodimers. They are the αβ T cells, γδ T cells, and γμ T cells (Hedrick et al. 1984; Chien et al. 1984; Brenner et al. 1986; Morrissey et al. 2021). All mammals appear to have αβ and γδ T cells, however only the marsupials and monotremes also have γμ T cells (Rast et al. 1997; Parra et al. 2007; Wang et al. 2011). The five TCR chains that make up these receptors, TCRα, TCRβ, TCRγ, TCRδ, and TCRμ, are encoded by the *TRA/D, TRB, TRG*, and *TRM* loci, respectively (Davis and Bjorkman 1988; Brenner et al. 1986; Parra et al. 2007, 2008). The conventional TCR chains, TCRα, β, γ, and δ, consist of a single variable (V) and a single constant (C) extracellular domain. The V domain is encoded by an exon that is assembled by V(D)J recombination during T cell development, by recombining the V, D, and J gene segments for β and δ chains and V and J gene segments for the α and γ chains. The genes encoding the conventional TCR are typically organized in a translocon fashion as [(V)n – (D)n – (J)n – C] or [(V)n – (J)n – C] (Davis and Chein 2003).

**Fig. 1:**
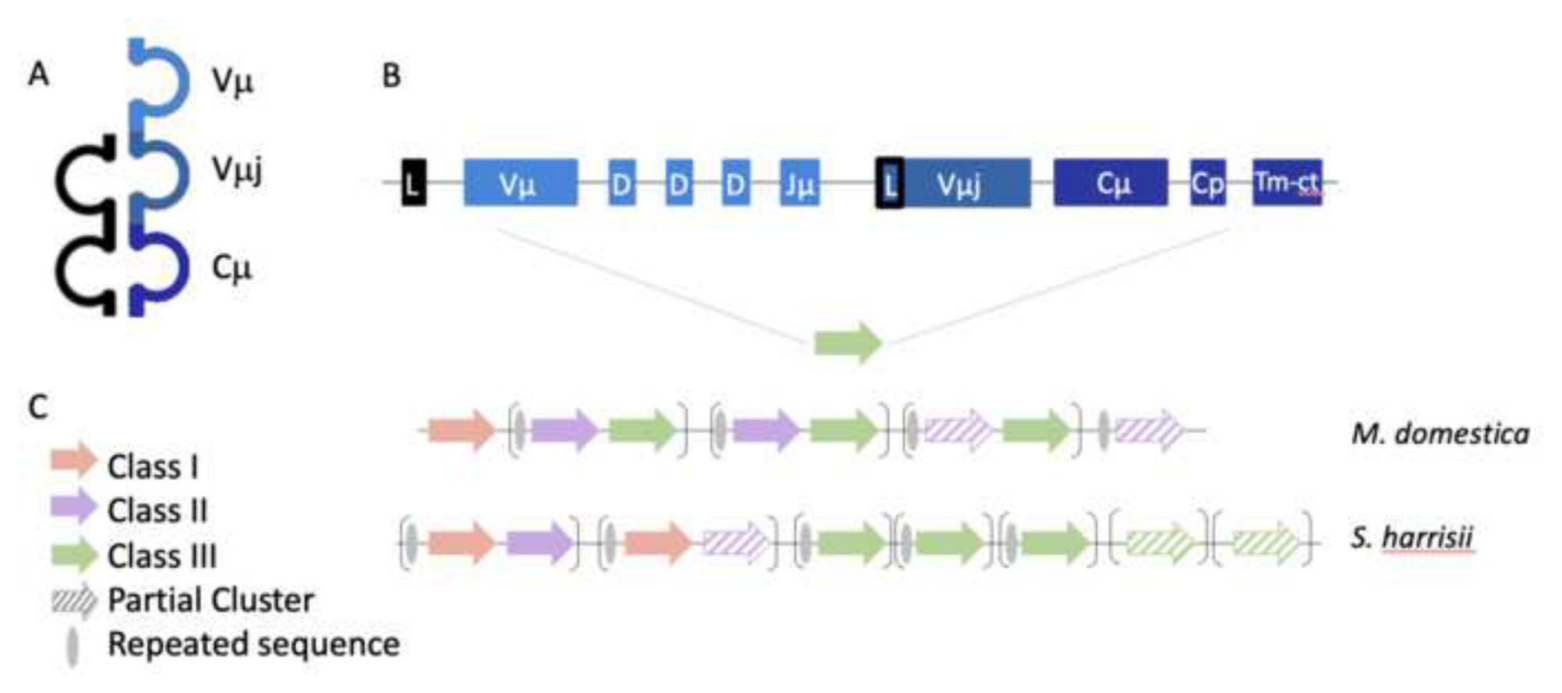
**A** Cartoon representation of the marsupial γμTCR, TCRγ shown in black and TCRμ in blue. **B** Representation of a full cluster [Vμ-Dμ-Jμ-Vμj-Cμ]. Gene segments shown in blue correspond to the TCRμ domain they encode; Vμ in light blue, Vμj in blue and Cμ in dark blue. **C** Comparative maps of the *M. domestica* and *S harrisii TRM* loci. Solid arrows indicate full clusters while dashed arrows indicate partial clusters. Transcriptional orientation is indicated by the direction of the arrow on each cluster. Arrows are not proportionate to actual cluster size. Brackets illustrate duplication patterns, and a gray ellipse is the predicted duplication start site.

The TCRμ chain used in the γμTCR is unconventional in that it has two V regions and a single C region as their extracellular domains (Fig. 1a, Parra et al. 2007). TCRμ appears to have evolved from TCRδ and its C region genes are most closely related to that of *TRD* (Parra et al. 2007, 2012a). In contrast, TCRμ V regions (Vμ) are most similar to antibody heavy chain V (VH) and structurally most similar to the VH domains of light-chainless antibodies found in camelids (Baker et al. 2005; Parra et al. 2007, 2008; Morrissey et al. 2021). The N-terminal V domain of the TCRμ chain is encoded by an exon assembled by recombination of Vμ, Dμ and Jμ gene segments to create a highly diverse, clonally specific repertoire. The second V domain, called Vμj is encoded by a pre-joined or germline joined exon, and is invariant between T cells clones (Parra et al. 2007). The gray short-tailed opossum, *Monodelphis domestica*, has been the primary model marsupial for studies of TCRμ (Parra et al. 2007). The opossum *TRM* locus is located on chromosome 3q, unlinked to the conventional TCR loci, and is organized as a series of tandem clusters: [Vμ – Dμ – Jμ - Vμj - C] [Vμ – Dμ – Jμ - Vμj - C] [Vμ – Dμ – Jμ - Vμj-C] etc. (Fig. 1b and c, Parra et al. 2007, 2008). There are six complete clusters and two partial clusters in the opossum, with the two partial clusters both lacking the Vμ and Dμ (Fig. 1c, Parra et al. 2007, 2008). Like conventional T cells, opossum γμ T cells develop in the thymus and are first detected around postnatal week 2 (Parra et al. 2007, 2009).

**Fig. 2:**
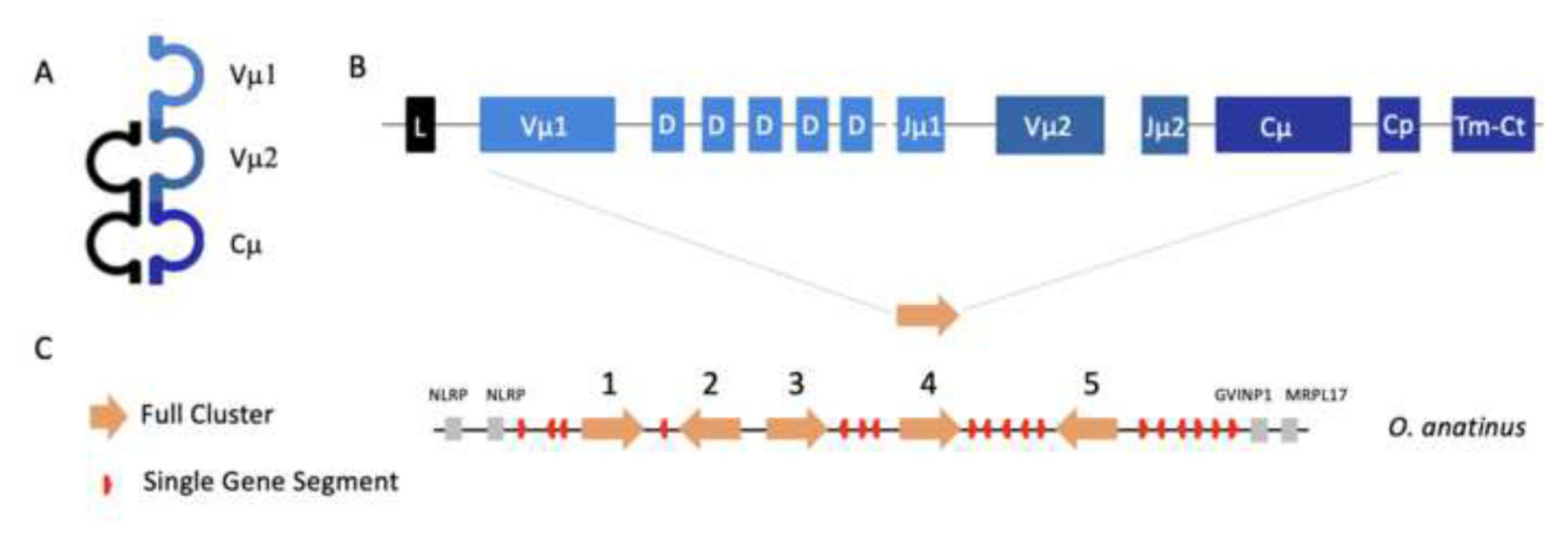
**A** Cartoon representation of the monotreme γμTCR, TCRγ shown in black and TCRμ in blue. **B** Representation of a full cluster [Vμ1-Vμ2-Cμ]. Gene segments shown in blue correspond to the TCRμ domain they encode; Vμ1 in light blue, Vμ2 in blue and Cμ in dark blue. **C** Summarized map of the *O. anatinus TRM* locus. Solid orange arrows indicate full clusters while red arrows indicate a single gene segment. Transcriptional orientation is indicated by the direction of the arrow on each cluster or gene. Arrows are not proportionate to actual cluster or gene size. Gray boxes indicate syntenic gene NLR family pyrin domain containing 10 (NLRP)

The TCRμ chains in a monotreme, the duckbill platypus, *Ornithorhynchus anatinus*, have a predicted structure similar to marsupial TCRμ, and are clearly orthologous (Fig. 2a, Wang et al. 2011). The N-terminal V domain, Vμ1, is encoded by recombination of a V with multiple D segments and a J that results in diverse V domains. The exon encoding the second V domain, Vμ2, is encoded by V and J gene segments however due to lack of D segments there is limited junctional diversity, mimicking the germline joined Vμj in the opossum (Fig. 2b, Wang et al. 2011). The presence of an orthologous *TRM* locus in both marsupials and monotremes supports it being present in the last common ancestor of all living mammals and, therefore, lost in the placental lineage (Wang et al. 2011).

In this study, we investigate the evolution of the *TRM* genes, comparing the locus organization, complexity, and content between two marsupial species and a monotreme.

## Materials and Methods

### Genomic sequences

The *Ornithorhynchus anatinus genome* assembly (mOrnAna1.pri.v4 reference Annotation Release 105, NC_041729.1) was searched to identify the *TRM* locus using previously published platypus sequences (Wang et al. 2011). The *Sarcophilus harrisii* (Tasmanian devil) genome assembly (mSarHar1.11, GenBank accession number GCA_902635505.1, NC_045426.1) was searched to identify the *TRM* locus by BLASTn using Vμ, Vμj and Cμ sequences identified in the *S. harrisii* transcriptome analyses. Genome assembly characteristics are in Supplementary Table 1.

### Transcriptome analyses

TCRμ transcripts in *S. harrisii* were identified by utilizing Vμ, Vμj and Cμ gene sequences from *M. domestica* to probe a transcriptome in a local database utilizing BLASTn (Parra et al. 2008). Open reading frames of the recombined V-D-J gene segments were searched to identify productive transcripts using SnapGene® software (from Insightful Science; snapgene.com). The outputs were analyzed for V and C regions based on conserved motifs and were subjected to BLASTp against the Genbank database. The *S. harrisii*, transcriptome assembly (PRJEB34650) accession numbers of utilized sequences are in Supplementary Table 2.

### Annotation and characterization

Non-TCR genes in the *S. harrisii* and *O. anatinus* genomes which flank the *TRM* locus were previously annotated in NCBI. BLASTn was utilized on all coding sequences against the GenBank database for annotation. Germline V, J and C gene segments were located using one or more of the following methods: 1) comparing cDNA to the *TRM* locus; 2) searching for conserved amino acid motifs; and 3) by identifying the recombination signal sequences. The D segments were identified using sequences which represent the V-D-J junctions by aligning transcripts to the genomic sequence and by searching for flanking recombination signal sequence (https://www.itb.cnr.it/rss/analyze.html). V gene nucleotide sequences corresponding to framework 1 through framework 3 were utilized in the identity matrix generated by *Sequence Demarcation Tool Version 1.2* (Muhire et al. 2014).

Previously, opossum *TRM* clusters were assigned to one of three classes (Parra et al. 2008). Newly annotated marsupial genes were assigned to the opossum classes if they have greater than 75% nucleotide identity. *O. anatinus* gene segments are annotated according to their location from the 5’ to 3’ end of the locus. Vμ1 genes were identified by presence of D segments following the V gene and by sequence identity if the V gene was not clearly in a cluster. Vμ2 genes were identified by the lack of D segments following the V gene and sequence identity to known Vμ2. *S. harrisii* gene segments within a cluster are numbered according to their location from the 5’ to 3’ end of the locus.

## Results

### The *TRM* locus in Australasian marsupials

Previous analyses of the *TRM* loci in multiple marsupial species, such as the bandicoot, revealed differences in the number of clusters as compared to the opossum, however, extensive comparisons of this locus in other marsupial species have not been performed. (Baker et al. 2005; Parra et al. 2008). To further investigate the evolution of the *TRM* loci, we compared two marsupial species, Tasmanian devil (*S. harrisii)* and opossum (*M. domestica)*, and one monotreme, the platypus (*O. anatinus)*.

In the opossum, each cluster falls into one of three classes based on sequence identity (Parra et al. 2008). Class I is comprised of single cluster, cluster 1, which does not share significant sequence identity with other clusters. Class II includes clusters 2, 4, 6 and 8, and class III includes clusters 3, 5 and 7 (Parra et al. 2008). The clusters in opossum alternate between class II and class III (Parra et al. 2008) and appear to have duplicated in tandem (Fig. 1c).

**Fig. 3:**
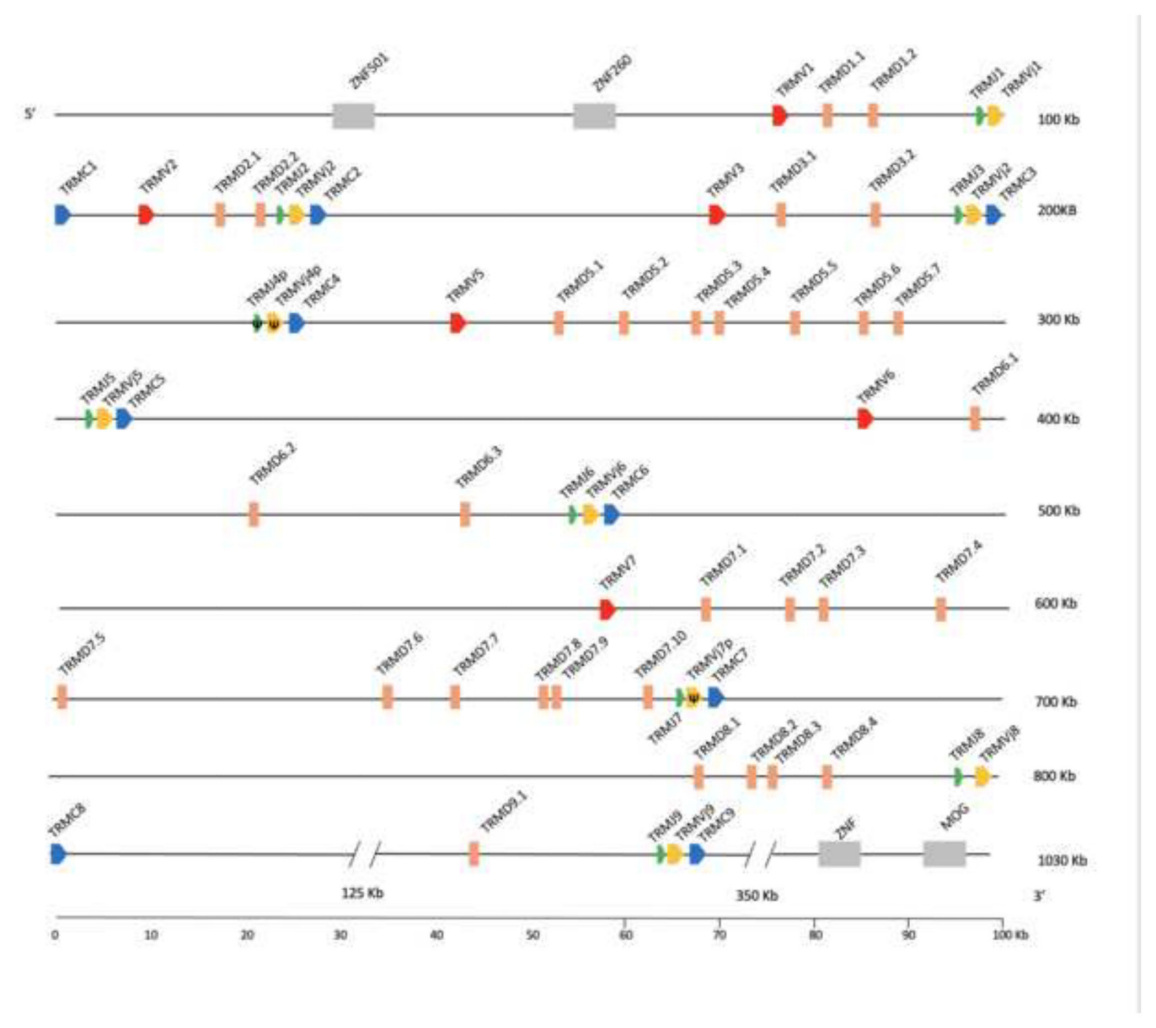
Map of the *S. harisii TRM* locus. *TRMV* (red), *TRMD* (peach), *TRMJ* (green), *TRMVj* (yellow) and *TRMC* (blue) gene segments are numbered by their corresponding location in order across the locus. Transcriptional orientation is indicated by the direction of the arrow on each gene segment. Arrows are not proportionate to the actual gene sizes. Gray boxes indicate syntenic genes. Presumptive pseudogenes are indicated with a Ψ

The devil *TRM* locus is on chromosome 1 and spans approximately 1Mb (Fig. 3). Nine clusters were identified, of which three are partial. Cluster 4 lacks the Vμ and Dμ gene segments (Figs. 1c and 3, Parra et al. 2008). Clusters 8 and 9 lack only the Vμ gene. The *TRM* loci of both marsupials are flanked by conserved, syntenic genes, including zinc finger protein genes at the 5’ end and myelin-oligodendrocyte glycoprotein (MOG) at the 3’ end. The opossum also has the speckle type POZ-like protein (PCIF1-like) gene between cluster 8 and MOG, which were not identified proximal to the devil *TRM* locus (Figs. 3, Parra et al. 2008).

**Fig. 4:**
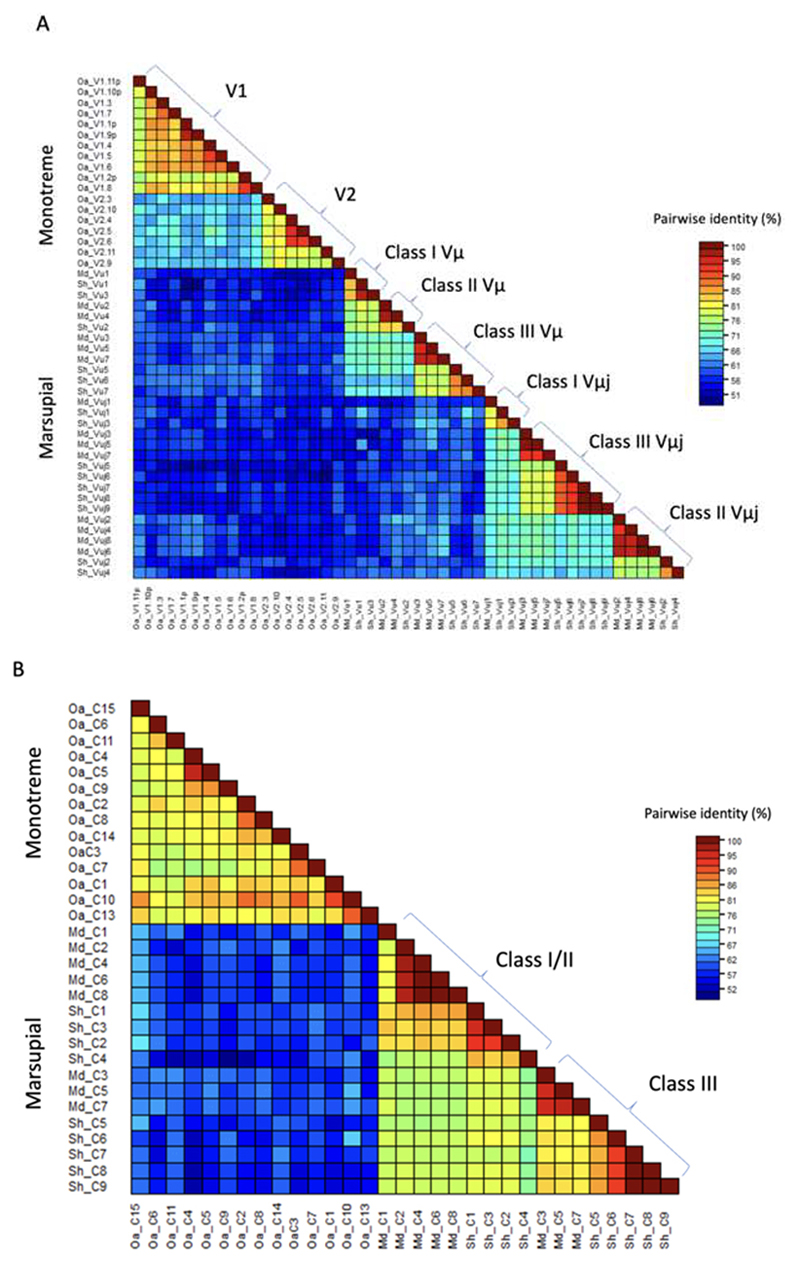
Identity matrix of non-eutherian *TRMV* (**A**) and *TRMC* (**B**) genes. Species are indicated on the left and gene classes are indicated on the right of the matrix

The three classes of *TRM* clusters found in the opossum are conserved in devil (Fig. 4). In the devil, clusters 1 and 3 are class I, clusters 2 and 4 are class II and clusters 5-9 are class III (Fig. 4). However, unlike the opossum, the *TRM* clusters in devil are a mix of tandem and singlet duplications (Fig. 1c). Upstream of some clusters in both marsupial species are repeated elements containing sequences resembling endogenous retroviruses and LINEs (Fig.1c). These repeats are upstream of the first cluster in the tandem pair. In the devil, where there are single cluster duplications, each duplicated cluster has its own upstream repeat. These observations are consistent with a mechanism of duplication involving non-homologous crossovers utilizing the repeat sequences.

Although the overall organization of the *TRM* locus varies between marsupial species, they all retain the cluster organization for the coding sequences. To investigate the origins of this organization we analyzed the only non-marsupial lineage that has TCRμ, the monotremes, specifically the platypus *O. anatinus* (Wang et al. 2011), a species separated from marsupials 180 million years ago (van Rheede et al. 2006).

**Fig. 5:**
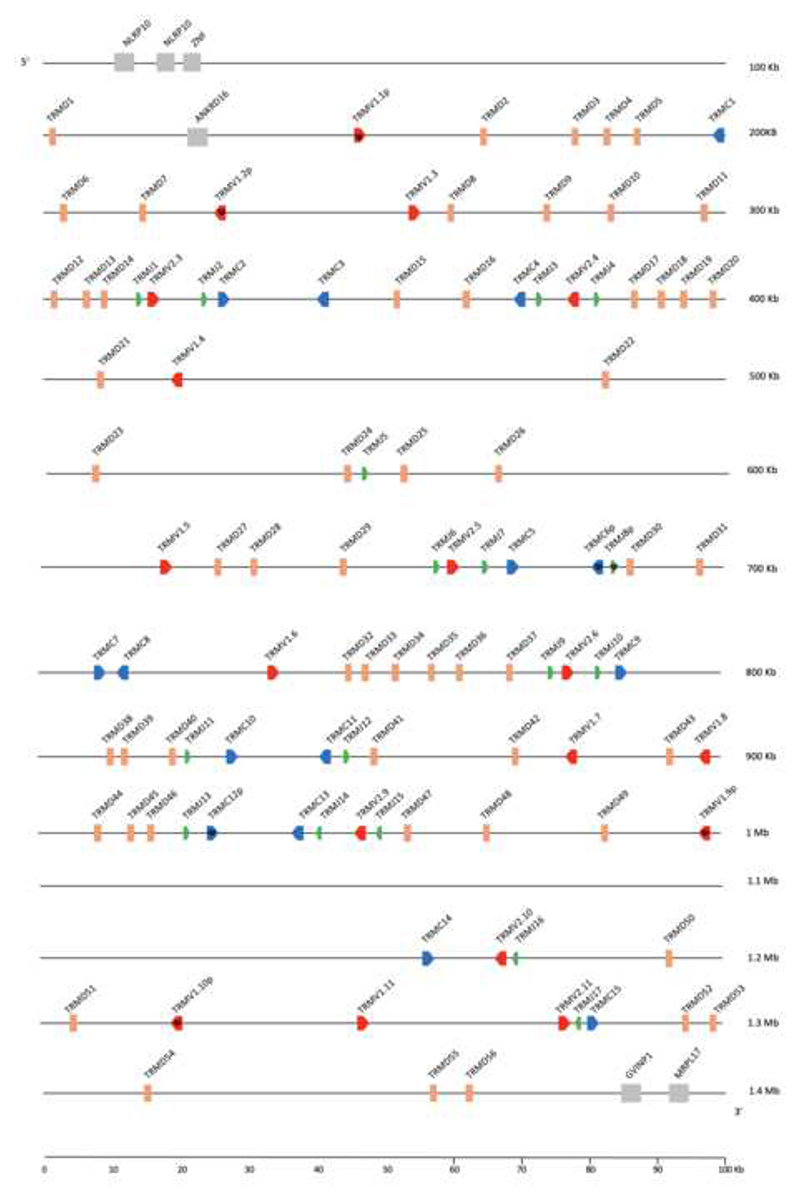
Map of *O. anatinus* locus. *TRMV* (red), *TRMD* (peach), *TRMJ* (green), and *TRMC* (blue) gene segments are numbered by their corresponding location in order across the locus. Vμ1 and Vμ2 genes are indicated. Transcriptional orientation is indicated by the direction of the arrow on each gene segment. Arrows are not proportionate to the actual gene sizes. Gray boxes indicate syntenic genes. Presumptive pseudogenes are indicated with a Ψ

The platypus *TRM* locus was located in the currently available platypus genome assembly using previously published transcripts (Wang et al. 2011). The platypus locus is on chromosome 2 and spans approximately 1.3Mb (Fig 5). There is not conserved synteny with the flanking genes found in marsupials. Rather the platypus *TRM* locus is flanked by NLR family pyrin domain containing 10 (NLRP10) and ZNF at the 5’ end, and interferon-induced very large GTPase 1-like (GVINP1) and mRNA-mitochondrial ribosomal protein L17 (MRPL17) at the 3’ end (Fig. 5). Unfortunately, we were unable to identify a clear *MOG* ortholog in the platypus genomic assemblies available, nor were we able to identify *NLRP* and *GVINP1* in either the current opossum or Tasmanian devil genomes.

Eighteen V gene segments were identified, of which 11 share identity with known Vμ1 gene segments. The Vμ1 in clusters were confirmed also by the presence of downstream D segments. The remaining 7 V segments shared identity with the known Vμ2 genes, which lack downstream D segments. There were fifteen C gene segments identified, five of which were located with the clusters and the remaining as singlets interspersed throughout the locus (Figs. 5 and 2c). The platypus genes do not correspond to any of the *TRM* gene classes in marsupials, sharing less than 64% nucleotide identity with their marsupial homologues (Figs. 4a and b).

There are five clear clusters in the platypus, composed of a [V1-D_N_-J1-V2-J2-C]. However, they are not in tandem and there are other *TRM* related gene segments interspersed throughout the locus (Fig. 5). It is noteworthy that the previously identified platypus TCRμ transcripts (Wang et al. 2011) appear to be encoded by gene segments with a cluster organization (not shown). This result is consistent with an ancestral cluster present in the last common mammalian ancestor which duplicated and diverged in sequence.

## Discussion

The conventional TCR chains and genes have remained remarkably conserved over most of jawed-vertebrate evolution (Rast et al. 1997). Where evolutionary plasticity does exist, it is primarily in the genes encoding the TCRδ chain. In several vertebrate lineages, including sharks, coelacanths, amphibians, rhynchocephalians, birds, crocodilians and monotremes, TCRδ chains can be found that use either conventional Vδ genes or antibody VH (VHδ) genes interchangeably (Criscitiello et al. 2010; Parra et al. 2010, 2012a, 2012b; Saha et al. 2014; Wang et al. 2020; Morrissey et al. 2022). Two extreme examples of the plasticity of TCRδ are: 1) the mammalian TCRμ chain that appears to have evolved from TCRδ early in mammals and is now only found in marsupials and monotremes (Parra et al. 2007; Wang et al. 2011) and 2) the evolution of a form of TCRδ, called NAR-TCR, in sharks that appears to be structurally analogous to TCRμ with three extracellular domains (Criscitiello et al. 2006). Having evolved independently, TCRμ and NAR-TCR have converged on a common structure. Another extreme example is the complete loss of the γδ T cell lineage by deletion of the *TRD* and *TRG* genes in squamate reptiles (Morrissey et al. 2022). The squamates are not a niche vertebrate lineage, as are other examples where components of the T cell lineage have been lost such as cod and anglerfish (Swann et al. 2020; Star et al. 2011; Malmstrøm et al. 2016). Rather, there are more than 10,000 squamate species that inhabit a broad range of ecosystems, from marine snakes to desert lizards.

The goal of this study was to examine the genomic architecture of the *TRM* loci across distantly related marsupials and one monotreme to gain insight into the evolution of this unique and unconventional locus. Genomic analyses revealed all marsupial genes can be categorized into three conserved classes, that predate marsupial speciation. However, dissimilarity within the organization of the *TRM* loci between marsupial species is consistent with lineage specific duplications, which may be related to the presence of repetitive sequences interspersed between clusters.

The marsupial TCRμ is the only known TCR with germline joined V genes (Parra et al. 2007). Fully or partially recombined genes in the germline are often found in Ig loci as has been identified in opossums, chickens, and sharks (Wang and Miller 2012; Reynaud et al. 1989; Dooley et al. 2006; Lee et al. 2000; Rumfelt et al. 2002). The Vμj in marsupials has recently been shown to perform a structural role within the γμTCR heterodimer, pairing with a nearly invariant Vγ (Morrissey et al. 2021). This finding supports thymic selection of invariant Vγ domains to pair with TCRμ in developing γμTCR (Parra et al. 2007; Morrissey et al. 2021).

The marsupial and monotreme *TRM* loci are clearly homologous (Wang et al 2011), however, the platypus *TRM* gene segments fall outside the three marsupial *TRM* classes, forming their own independent clade. This likely is due to the long evolutionary time separating the monotremes from the marsupials. Likewise, conserved synteny has not been maintained between the divergence of monotremes from marsupials, approximately 180 mya, while it has between American opossums and Australian marsupials that diverged less than 80 mya (Meredith et al. 2008). This result is surprising given that it is generally typical for TCR loci to share conserved syntenic flanking genes, even across comparable evolutionary distances (Morrissey et al. 2022). This is consistent with chromosome recombination since monotreme and marsupial divergence and possibly the translocation of the *TRM* locus as has occurred with chicken *TRD* locus creating a second locus (Parra et al 2012b).

In the platypus the exon encoding the Vμ1 domain is assembled from V, D, and J gene segments, identical to the Vμ domain in marsupials. The Vμ2 domain in platypus correlates to the Vμj in opossum, however, is not germline joined but assembled by recombination of a V and J (Wang et al. 2011). The lack of D segments results in limited diversity in Vμ2, mimicking the germline joined Vμj in marsupials. The short Complementary Determining Region-3 loop encoded by Vμj, would be consistent with the absence of the D segments between Vμ2 and Jμ2, prior to the divergence of the monotremes from the therian mammals (marsupials and eutherians). Previous analyses of the platypus *TRM* germline sequences were based on a highly fragmented and partial genome assembly (Wang et al. 2011). The analysis of the updated platypus genome confirms that the germline joined Vμj gene found in all marsupials is not present in the platypus and, therefore, evolved after the separation of monotremes from the therian mammals, most likely by retrotransposition (Parra et al. 2007).

Models for the origins of TCRμ have emerged through comparative genomics of both mammals and non-mammals (Parra et al. 2012a; Wang et al. 2020). In frogs, for example, the TCRδ chains that use VHδ are inverted relative to the rest of the *TRA/D* locus (Parra et al. 2010). In galliform birds there is both a conventional *TRA/D* locus encoding the TCRα and TCRδ chains, along with a second, unlinked locus that encodes a TCRδ chain that uses VHδ for assembling the V region (Parra et al. 2012b). Monotremes, so far, remain the only known vertebrates that have both TCRδ that use VHδ along with a separate TCRμ, and probably represent the closest to the last common ancestor in mammals. The organization of the platypus *TRM* locus, with evidence of having a cluster organization, is consistent with previous models of the origin of TCRμ through duplication and divergence of the *TRD* locus (Parra et al. 2012a; Wang et al. 2020).

Many unanswered questions regarding the γμ T cell remain, not the least of which is their effector functions in an immune response. In addition, the nature of ligand recognition and binding by the γμTCR, and the apparently analogous shark NAR-TCR, remains unknown. Yet these questions may need to be answered before we can understand why the largest and most successful group of mammals, the eutherians, lost the γμ T cell lineage.

## Supplementary Material

Supplementary Table 1

Supplementary Table 2
