# Supplementary material for: Comparative genomics of the T cell receptor μ locus in marsupials and monotremes
Source: Immunogenetics. Author manuscript; Available in PMC 2024 Mar 20. (PMC7615758; doi:10.1007/s00251-023-01320-w)
Supplement: Supplementary Table 1 [file EMS194722-supplement-Supplementary_Table_1.pdf]

Supplementary Table 1. Genome assembly characteristics.

Metric	*S. harrisii*	*M. domestica*	*O. anatinus*
Scaffold N50	611.3Mb	59.8Mb	83.3Mb
Contig N50	62.3Mb	108kb	15.1Mb
**BUSCO analysis**			
Complete	95.50%	91.60%	92.3%(S+D)
Single-copy	94.50%	90.30%	91.20%
Duplicated	1.00%	1.20%	1.10%
Fragmented	0.90%	2.00%	1.40%
Missing	3.60%	6.40%	6.30%
