# Supplementary material for: Comparative genomics of the T cell receptor μ locus in marsupials and monotremes
Source: Immunogenetics. Author manuscript; Available in PMC 2024 Mar 20. (PMC7615758; doi:10.1007/s00251-023-01320-w)
Supplement: Supplementary Table 2 [file EMS194722-supplement-Supplementary_Table_2.pdf]

**Supplementary Table 2. Accession numbers of identified TCRμ transcripts in *S. harrisii*.**

| Accession | Length | Identified genes |
|---|---:|:---:|
| **DN4741_c0_g1_i1** | 1465 | TRMV3, TRMD3.1, TRMJ3, TRMVj3, TRMC3 |
| **DN250425_c0_g1_i1** | 877 | TRMV5, TRMJ5, TRMVj5 |
| **DN10998_c1_g1_i4** | 1564 | TRMV5, TRMJ5, TRMVj5, TRMC5 |
| **DN109828_c1_g5_i1** | 1126 | TRMV5, TRMJ5, TRMVj5, TRMC5 |
| **DN68022_c0_g6_i1** | 912 | TRMV5, TRMJ5, TRMVj5, TRMC5 |
| **DN68022_c0_g6_i2** | 796 | Class III TRMV, TRMJ, TRMVj, TRMC |
| **DN2685_c1_g1_i13** | 2050 | TRMV5, TRMJ5, TRMVj5, TRMC5 |
| **DN969_c0_g1_i6** | 1649 | TRMV5, TRMJ5, TRMVj5, TRMC5 |
| **DN969_c0_g1_i17** | 882 | Class III TRMV, TRMJ |
| **DN969_c0_g1_i10** | 2233 | TRMV5, TRMJ5, TRMVj5, TRMC5 |
| **DN969_c0_g1_i12** | 2048 | TRMV5, TRMJ5, TRMVj5, TRMC5 |
| **DN9495_c0_g2_i2** | 925 | Class III TRMV, TRMJ, TRMVj |
| **DN9495_c0_g2_i6** | 686 | Class III TRMV, TRMJ |
| **DN9495_c0_g2_i9** | 865 | TRMV5, TRMJ5, TRMVj5 |
| **DN9495_c0_g2_i3** | 626 | TRMV5, TRMJ5 |
| **DN105161_c0_g2_i1** | 381 | TRMV7, TRMJ7, TRMVj7 |
